# Identifying recommendations to improve therapy-led management of complex regional pain syndrome in England

**DOI:** 10.1177/20494637251389063

**Published:** 2025-10-25

**Authors:** Jessica Coggins, Candida McCabe, Nicola Walsh, Jennifer Pearson, Catherine Rolls, Alison Llewellyn

**Affiliations:** 1 1981 School of Health and Social Wellbeing, University of the West of England, Bristol, UK; 2 Centre for Education and Research, Dorothy House Hospice, Winsley, Wiltshire, UK; 3 School of Psychological Sciences, University of Bristol, Bristol, UK; 4 1984 Hand Therapy Department, University Hospitals Bristol and Weston NHS Foundation Trust, Bristol, UK

**Keywords:** complex regional pain syndrome, therapy-led management, survey, qualitative interviews, recommendations

## Abstract

**Background:**

Complex Regional Pain Syndrome (CRPS) is a distressing pain condition that can lead to significant burdens for individuals and society. A small number of specialist CRPS-practice services exist, but access is limited; most patients are managed in local hospitals or community settings. In a series of phases, we sought to understand the needs and perspectives of therapy practitioners across the care pathway in England and to identify recommendations to improve therapy-led management of CRPS.

**Methods:**

In phase 1, we disseminated an e-survey to physiotherapists, occupational therapists and hand therapists across a range of settings. Phase 2 comprised semi-structured interviews with therapists and patients. Online stakeholder events (phase 3) were convened to review findings and prioritise suggestions to enhance CRPS care.

**Results:**

Seventy-seven therapists responded to the e-survey and 31 semi-structured interviews were conducted (n = 10 patients, n = 9 therapists from specialist CRPS-practice services, n = 12 therapists from other settings). N = 11 therapists and n = 5 patients participated in the stakeholder events. Findings indicated pathways of care are complex, but similarities exist in therapy approaches across settings, albeit with longer, more frequent appointments in specialist CRPS-practice services. Recommendations to improve CRPS management included: additional education provision for therapists (including better access to CRPS ‘experts’), CRPS education for other clinicians, streamlining of patient pathways, and improving patient information.

**Conclusions:**

This work highlights opportunities to improve therapy-led care for people living with CRPS. Findings will be valuable in informing UK clinical guidelines and strengthening initiatives to enhance support for therapists.

## Introduction

Complex Regional Pain Syndrome (CRPS) is a highly distressing and disabling chronic pain condition, predominantly arising following limb injury or surgery. It usually begins in a distal extremity and is characterised by pain that is disproportionate to what would be typical for the type of tissue trauma experienced.^1^ Some cases of CRPS are also spontaneous, with no identifiable precipitating trauma.^2^ Diagnosed using the empirically derived Budapest Criteria, CRPS is associated with changes in the limb including swelling, changes in hair and nail growth, skin temperature and skin colour, as well as severe, persistent pain.^3^ People with the condition may also experience extreme hyperalgesia and allodynia, as well as altered body perception involving abnormal sensations and distorted mental representations of their affected limb.^1^ It is considered a complicated multifactorial disease, with experts suggesting it may encompass subtypes as well as differences in severity between individuals.^4^ European population studies estimate a CRPS incidence of 26/100,000 person-years.^5^

CRPS symptoms can be quite common after fracture (e.g. estimates suggest CRPS is reported in up to 25% of distal radius fractures^6^), and most cases resolve spontaneously in the first few months; however, for a small number of patients, symptoms do not respond to usual care and develop into chronic CRPS. There is no cure for chronic CRPS, and retrospective studies report symptoms persist for between 22% and 64% of patients ≥3 years after diagnosis.^7,8^ Although this equates to a relatively small number of people, CRPS which fails to respond to usual care causes significant physical and emotional burden to individuals and health services. Furthermore, long-term work incapacity has been observed in two-thirds of cases.^9^

Comprehensive United Kingdom (UK) guidelines for CRPS have been published by the Royal College of Physicians; based on expert consensus they advocate diagnosis via the Budapest Criteria^3^ and provide recommendations for Physiotherapists and Occupational Therapists including prompt diagnosis and early referral for therapies that encourage limb movement and use from the outset.^2^ However, a 2017 Freedom of Information request revealed no CRPS care pathway or agreed initial CRPS management exists in 82% of English National Health Service (NHS) Trusts with CRPS-relevant services.^10^ A survey of clinicians working in the field of CRPS further identified that over half of UK respondents reported difficulty in recognising the signs and symptoms of CRPS, highlighting a lack of awareness of the condition by healthcare professionals.^11^

A small number of NHS services exist in England in which patients with CRPS are offered longer and more frequent appointments than in most settings, and where staff have recognised experience, and particular interest, in CRPS. For the current study, these were defined as ‘specialist CRPS-practice services’. However, it is impractical, too resource intensive, and not always clinically appropriate, for all individuals with CRPS to be referred to these services; thus, only a very limited number of people with more severe and refractory CRPS are seen by these services and can benefit from this specialist care. Therapists in non-specialist settings (i.e. those we did not define as ‘specialist CRPS-practice services’), therefore, require the ability to recognise CRPS and the knowledge and confidence to treat, or to appropriately refer on. The low incidence of CRPS can mean a therapist may only see a handful of cases per year, pathways of care are often considered less than optimal, and published standards are frequently not followed.^10^ Understanding the needs, perceptions and experiences of therapists and people with CRPS was considered important in developing recommendations to enhance care. To address this problem, our study aimed to: (1) better understand the needs and perspectives of therapy practitioners working in non-specialist and specialist CRPS-practice services across the care pathway in England; (2) identify any differences in therapy practices within, and outside of, specialist CRPS-practice services; (3) explore patient and therapist experiences in specialist CRPS-practice services; and (4) work with stakeholders to agree priorities for change and which are feasible for delivery in current NHS services.

## Methods

Adopting a mixed-methods sequential exploratory design,^12^ the study consisted of a pre-work scoping exercise, followed by three phases of research: (1) electronic survey (e-survey), (2) qualitative interviews, and (3) data integration and stakeholder events. All aspects of the study were overseen by an experienced project management group led by the Chief Investigator (CI): AL, with other members comprising: CM, NW, JP, CR, JC, and a UK patient contributor. Day-to-day activities were conducted by a Research Associate (RA): JC. Additional oversight was provided by an independent steering committee whose membership included a patient contributor not otherwise involved in the study.

### Pre-work: Scoping exercise to define specialist CRPS-practice services

An initial scoping exercise was undertaken in the form of an online survey sent to members of The CRPS Network. This UK network comprises clinicians and researchers with interest and expertise in CRPS and of which AL and CM are members. Responses from network members identified 12 specialist CRPS-practice services in England.

### Phase 1: e-survey

Informed by a prior review of the literature,^13^ a national cross-sectional e-survey of physiotherapists, occupational therapists, and hand therapists who worked, or had worked, with people with CRPS in England was conducted between October and December 2021, led by NW and AL. The survey was hosted on the Qualtrics survey platform and disseminated via the Chartered Society of Physiotherapy, Royal College of Occupational Therapists, British Association of Hand Therapists, Council for Allied Health Professions Research, and via the study’s own social media. Participants provided informed consent at the start of the survey by agreeing to a set of statements before they could proceed. Based on a prior e-survey conducted by members of the study team, a survey response of n ≈ 100 was anticipated. Data were descriptively analysed (by JC and CR). Questions sought to determine current provision and practice in England and therapists’ understanding of CRPS, as well as the formal and informal training they had received (respondents were invited to self-define ‘formal’ and ‘informal’ training as a free text response). Questions also identified published guidelines used by respondents and their perceived barriers or facilitators to CRPS management.

### Phase 2: Qualitative interviews

Three sets of semi-structured individual qualitative interviews were conducted, by telephone or online via Microsoft Teams, to explore individuals’ experiences of receiving, or delivering, care for people with CRPS. The study aimed to recruit n = 8-12 therapists from specialist CRPS-practice services; 12–16 therapists from other settings and n = 20 patients; however, the final sample size was pragmatically determined by the number of responses received to the invitations to participate. All interviews were arranged and conducted by JC, with oversight by AL as follows.

• In interview set 1, patients with CRPS were approached by email from the Administrator of the UK CRPS Registry. Sampling was consecutive starting with those patients who had most recently joined the Registry.
• Interview set 2 comprised therapists from specialist CRPS-practice services, as determined by the prior scoping exercise. Therapists in these centres were approached by members of the CRPS Network UK or were directly approached by an email from the study team (where consent to be re-contacted had been provided by participants in the prior scoping exercise or national e-survey).
• For interview set 3, therapists working outside of specialist CRPS-practice services were identified from an opt-in question in the prior national e-survey and invited to participate by email. Purposive sampling within this group aimed to ensure representation from different therapy professions and settings. Interview participants were emailed a comprehensive information sheet in advance, including the contact details of the CI and RA. Consent was received verbally at the start of each interview with participants joining from a location of their choice.

Topic guides for the qualitative interviews were compiled by members of the research team, which included a patient contributor (CC). Pilot interviews with patients and therapists were also conducted prior to data collection. All interviews were audio-recorded, transcribed verbatim, anonymised and uploaded to QSR NVivo 1.6.2 software for analysis. Data were analysed within a critical realist paradigm, thereby accepting participants’ reported realities but recognising these are contextualised within human practices and experiences.^14,15^ Braun and Clarke’s phases of thematic analysis were applied: reading and familiarisation; coding; searching for themes; reviewing themes; defining and naming themes; and finalising analysis.^14^ Data analysis of transcripts from interview sets 1 and 2 was conducted by JC and interview set 3 by AL. Analysis was considered complete when the generated themes were deemed to present sufficient richness in relation to the aims of the study.^16^ Methodological oversight of interviews and analysis was provided by CM and JP.

### Phase 3: Data integration and stakeholder events

Integration of data from the cross-sectional e-survey and interviews was conducted by AL and JC, informed by a triangulation protocol for qualitative health research in which commonalities between the data sets were identified.^17^ Using this method, suggestions to enhance care were identified from themes generated in the qualitative interviews with therapists and patients, and from individuals’ responses to the e-survey. These suggestions were presented by AL to stakeholders (patient representatives and therapists) during two, two-hour, online workshops using Microsoft Teams. Following the guidance of McMillan, King and Tully,^18^ we adapted the Nominal Group Technique, using the ranking and discussion phases to prioritise the suggestions. Participants were shown the suggestion items in an arbitrary order and asked to rate each on a 5-point scale, where 1 = ‘not important at all’, and 5 = ‘extremely important’. Items were then rated again in terms of perceived feasibility to change, where 1 = ‘very difficult to change’, and 5 = ‘very easy to change’​. A list of items for potential inclusion in a guidance document/resource pack was similarly compiled and presented and participants were asked to give their opinion of the helpfulness of each (1 point = ‘not at all helpful’, 5 points = ‘extremely helpful’). Finally, participants were asked to rate items in an additional ‘wish list’ generated by the interview data, where items scoring 1 point were considered ‘not useful at all’ and items with 5 points were considered ‘extremely useful’. Following ranking, aggregated results were presented by JC and participants were invited to share their reflections. The stakeholder meetings were hosted by AL and JC, with recordings retained as field notes to inform the reporting of findings.

Ethical approval was received for the e-survey from the University of the West of England Faculty of Health and Applied Sciences Research Ethics Committee (reference: HAS.21.07.173). Ethical approvals for the qualitative interviews and stakeholder workshops were received from the London–Brent NHS Research Ethics Committee (REC reference 21/PR/1763) and University of the West of England Faculty of Health and Applied Sciences Research Ethics Committee (reference: HAS.22.02.074).

## Results

### Phase 1: e-survey

The online survey of therapists who work with, or have worked with, people with CRPS in England received n = 77 eligible responses. Seventeen additional responses were excluded as they did not meet the eligibility criteria, for example, podiatrists or therapists working outside England. As shown in Table 1, therapy professions were represented by physiotherapists, hand therapists, and occupational therapists. Respondents’ work settings included specialist pain clinics, general outpatients, lone/small units, specialist hand units, and private clinics.

**Table 1.** Demographic characteristics of survey respondents (n = 77).

Characteristic	n =	%
Profession		
Hand therapist	6	7.8%
Occupational therapist	15	19.5%
Occupational therapist *and* hand therapist	8	10.4%
Physiotherapist	43	55.8%
Physiotherapist and hand therapist	5	6.5%
NHS Band (Grade)		
5	1	1.3%
6	21	27.3%
7	33	42.9%
8	19	24.7%
N/A	3	3.9%
Department/Setting		
General outpatient department – large teaching hospital	8	10.4%
General outpatient department – regional hospital	11	14.3%
Lone/small unit	4	5.2%
Other (private/rehab hospital)	10	13.0%
Pain clinic	18	23.4%
Specialist hand unit	26	33.8%

Over 90% of respondents reported seeing fewer than three patients with CRPS per month and whilst 68% were aware of guidelines for the management of CRPS, only 32% reported having a defined CRPS management pathway in their service. Respondents self-defined ‘formal’ training as including further education programmes, CRPS or profession-specific conferences/courses, and in-service training with ‘informal’ training primarily including self-directed learning, discussions with colleagues, observations, and webinars. Although 10% of therapists reported having received formal training about CRPS, just over half of all respondents (55%) had only received informal training and 12% had received no training at all about the condition. Despite this, most therapists (76%) were confident in diagnosing and describing CRPS. Detailed results are presented in Table 2. CRPS management approaches reported to be used by respondents are presented in Figure 1.

**Table 2.** Responses to online survey (n = 77).

Question	Response options				
How much do you know about CRPS as a condition?	Nothing at all	A little	A moderate amount	A lot	A great deal
	0 (0.0%)	5 (6.5%)	46 (59.7%)	20 (26.0%)	6 (7.8%)
Confidence in diagnosing CRPS	Extremely unconfident	Somewhat unconfident	Neither confident nor unconfident	Somewhat confident	Extremely confident
	4 (5.2%)	9 (9.1%)	7 (9.1%)	51 (66.2%)	8 (10.4%)
Confidence in describing CRPS to patients	Extremely unconfident	Somewhat unconfident	Neither confident nor unconfident	Somewhat confident	Extremely confident
	1 (1.3%)	7 (9.1%)	10 (13.0%)	51 (66.2%)	8 (10.4%)
Training received about CRPS	Formal training	Informal only	Both formal and informal	None received	
	8 (10.4%)	42 (54.5%)	18 (23.4%)	9 (11.7%)	
Approx. no of CRPS patients seen per month by the respondent	Fewer than 1	1–2	3–5	6–10	>10
	43 (55.8%)	28 (36.4%)	5 (6.5%)	1 (1.3%)	0 (0.0%)

**Figure 1.**
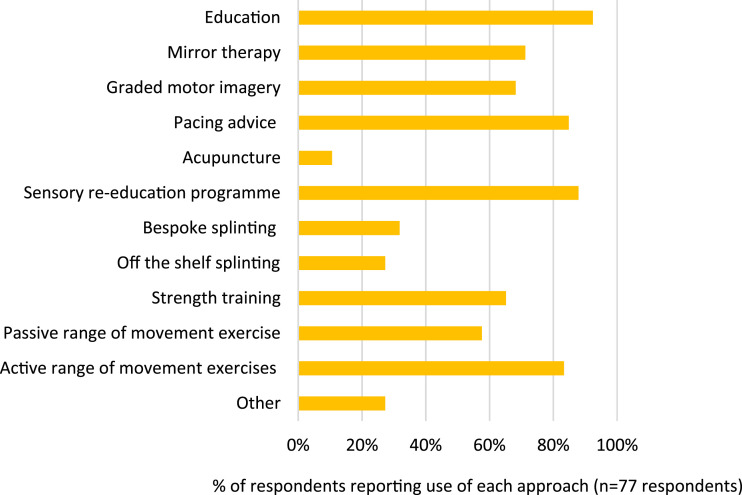
Reported therapy approaches for CPRS delivered in secondary/community care in England.

Included in the survey was a free text question which asked respondents to cite their perceived challenges to CRPS management that existed in their organisation or service. The most frequently reported factors were: lack of time/low staffing; lack of availability/frequency of follow-up appointments; low levels of knowledge/experience/understanding of CRPS by other healthcare professionals; and delayed diagnosis into service due to lack of understanding/awareness. Reported facilitators to good practice included education, awareness of treatment options, good communication, early diagnosis and intervention, and the ability to refer for specialist treatments when needed.

### Phase 2: Qualitative interviews

Individual, qualitative, semi-structured interviews were completed with n = 10 patients, n = 9 therapists working in specialist CRPS-practice services, and n = 12 therapists working outside specialist CRPS-practice services. Table 3 gives the demographic characteristics of these samples.

**Table 3.** Demographic characteristics of interview participants.

	Patient interviews	Therapist interviews	
	Interview set A	Interview set B: Therapists working in specialist CRPS-practice services	Interview set C: Therapists working outside specialist CRPS-practice services
Sample size	n = 10	n = 9	n = 12
Mean age (yrs)	53.4	44.2	45.8 (**n)
Age range (yrs)	40–66	30–55	35–54 (**n)
Ethnic group (*n)			
White	9	7	11
Mixed/multiple ethnic group	-	1	-
Other, not specified	1	1	-
Not reported	-	-	1
Assigned sex at birth (*n)			
Female	8	5	9
Male	2	4	2
Not reported	-	-	1
Mean interview time (mins)	58:49	55:08	48:36
Participants’ professions	N/A	Physiotherapist n = 6	Physiotherapist n = 6
		Occupational therapist n = 3	Occupational therapist n = 3
			Physiotherapist/hand therapist n = 1
			Occupational therapist/hand therapist n = 1
			Hand therapist n = 1

*n = number of participants, **n = responses received from 11 of 12 participants.

The four main themes generated by the qualitative analysis were: (1) pathways of care are complex, (2) therapists want education to feel confident and connected, (3) need for wider information, education, and understanding of CRPS, and (4) flexibility and goals in specialist CRPS-practice services. Illustrative quotes relating to each theme are given below.^1^

#### Pathways of care are complex

Consistent with the survey findings, which indicated a scarcity of defined pathways for CRPS management, an overarching theme generated by the interviews was that pathways of care are not straightforward or optimal. Therapists described their frustration that this lack of clarity could have deleterious effect on timely assessment and treatment:

“we've lost an overview of the local pathway, so we're not quite sure where patients are ending up, and where they're starting, even” (T)

So, I think it is probably slow, it is probably a long path for a patient to get to have a specialist service (SP)

Patients similarly reported disappointment in the time taken to receive a diagnosis and poor experiences in accessing care:

“I’ve had CRPS twenty-two years next month, but it took ten years to be diagnosed.” (P)

“The fact that GPs didn't know what was going on, even though they had a diagnosis, already, a confirmed diagnosis, they kept misdiagnosing.” (P)

“….it shouldn’t have been up to me as a patient to go to my GP and say I want to go there, can you send me there and for him to then argue with me.” (P)

A sub-theme further highlighted how challenges arising from systemic factors affect care. As was found in the e-survey, therapists working outside specialist CRPS-practice services reported concerns about inadequate staffing, limited clinical time with patients, and difficulties in accessing specialist teams, with referral to pain services and psychology considered particularly important:

“So, we have a significant increase in our referral numbers, and we haven't really had a relative increase in staffing.” (T)

… better access to the pain team and clinical psychology services would be our biggest hindrance at the moment… the waiting times for clinical psychology support are huge. Well over a year in our trust. (T)

“My feeling is that psychology is really important because invariably there'll be a psychological component that's huge.” (T)

#### Therapists want education in order to feel confident and connected

The interview data evidenced how therapists working outside of specialist CRPS-practice services can feel isolated and lack confidence. They reported wanting to better understand how to manage CRPS, particularly if patients were not progressing:

If there is no one else to help you, it’s really challenging. (T)

“We deskill very quickly if we're not seeing… if we're only seeing maybe one or two a year, then probably our skills aren't quite as good as they should be.” (T)

Whilst many therapists had made independent, informal, efforts to update their learning, their desire for education, support, and advice from clinicians with specialist expertise in CRPS was commonly reported:

“I’d want to be trained up by [an experienced clinician] who is aware of Complex Regional Pain Syndrome… also aware of what would be best practice, but how we can filter that down to establishments, such as this organisation here… I would love to have an all singing, all dancing, this is what Gold Standard care looks like.” (T)

[Ideal training and education] would be led by people who see CRPS on a normal day to day basis, who have the knowledge and expertise. It would cover identification… but then it would go through treatment, modalities… (T)

It was also suggested that improving education alongside the use of objective outcome measures might improve referral processes and access to specialist services:

“We’ve only got our outcome measures that we use within our specialisms… and when is it appropriate to say right, enough’s enough, we’re not making a big enough difference here… so maybe a set of core outcome measures that might be helpful in that decision making process (T)

#### Need for wider information, education, and understanding of CRPS

Patients and therapists both reported a need for wider education beyond for those already directly delivering CRPS care. Interview participants highlighted the importance of having accurate and appropriate patient information about CRPS available from the outset. Therapists similarly endorsed the need for GPs and non-specialist therapists to better understand the condition and for better education to be provided to other clinical staff, including surgical teams, who commonly see people with CRPS after the onset of the condition:

“I do feel that a lot of the GPs I see I probably know more about my disease than they do. Which is very frustrating.” (P)

“It's very, very common to find that patients have got a very poor understanding of their injury or the condition and what's been said to them” (SP)

#### Flexibility and goals in specialist CRPS-Practice services

Data from both the prior online survey and from the interviews indicated that therapy approaches delivered in specialist CRPS-practice services, and in other settings were broadly similar (see Figure 1 where therapy approaches from survey respondents are reported). However, notable exceptions were the additional provision of hydrotherapy; streamlined or embedded access to psychology services; and the use of cognitive multisensory rehabilitation (therapies in which patients are guided to try to ‘find’ normal sensations in their CRPS-affected limb) that were offered only in some specialist CRPS-practice services. Interview participants working in specialist CRPS-practice services also reported they perceived the aims and goals of their services to be different from other settings, citing a greater focus on function, rather than pain reduction:

“We always say that we are not a curative service, so one of the goals is to try to help people with CRPS live or improve their quality of life.” (SP)

“So, it's about carving out their future rather than say a more general pain management.” (SP)

Furthermore, therapists working in specialist CRPS-practice services more commonly reported working within multidisciplinary teams and having more flexibility regarding appointment times and duration.

“And I think in terms of how often we see them, that is generally a decision that’s made by us as therapists and the patient as well, so it’s often a combined approach to that sort of time frame.” (SP)

“So, new appointments are an hour, follow-ups are forty minutes, … how many times? It’ll vary, it’ll vary quite considerably” (SP).

A sub-theme generated from the patient interviews, all of whom had received care from specialist CRPS-practice services, included feeling reassured by a perception of greater understanding of their condition in those centres, and gratitude about being able to access longer appointments:

“It was great being with therapists that just understood everything that you were saying so quickly and so easily” (P)

“That essentially saved my life, I think, really. The symptoms have been manageable since.” (P)

However, the distances they sometimes had to travel to attend specialist CRPS-practice services and the time taken to attend multiple appointments were highlighted as barriers to care.

### Phase 3: Stakeholder workshops

Via online events, integrated findings from the online survey and interviews were presented to two separate heterogeneous cohorts comprising a total of 11 therapists (five from specialist CRPS-practice services) and five patient representatives (see Table 4). Adapted nominal group techniques were applied to (1) prioritise the most important of the suggestions to enhance care, (2) consider which of these suggestions would be most feasible to achieve, (3) prioritise those items considered most helpful for inclusion in a guidance document/resource pack, and (4) rank items from amongst a ‘wish list’ of other initiatives generated from the interview data. Findings, with individual item scores, are presented in Figure 2.

**Table 4.** Stakeholder group participants.

	Stakeholder group 1	Stakeholder group 2
Total participants	n = 10	n = 6
Therapy professionals	n = 7 (6 female, 1 male)	n = 4 (1 female, 3 male)
	Physiotherapist n = 3	Physiotherapist n = 2
	Occupational therapist n = 2	Occupational therapist n = 1
	Occupational therapist/hand therapist n = 1	Hand therapist n = 1
	Hand therapist n = 1	
	(n = 2 of the above were from specialist CRPS-practice services)	(n = 3 of the above were from specialist CRPS-practice services)
Patient contributors	n = 3	n = 2

**Figure 2.**
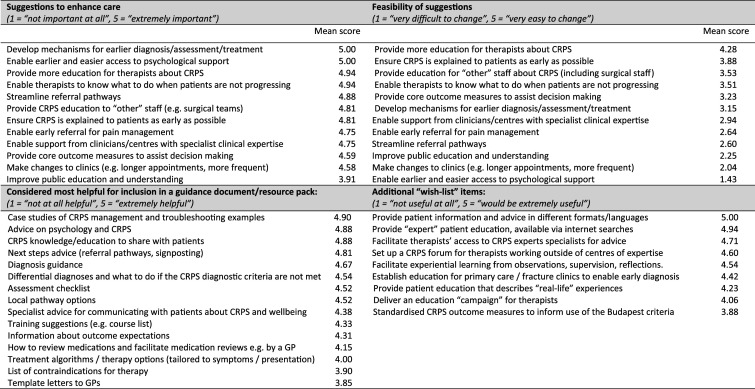
Priorities identified by heterogeneous stakeholder groups; mean scores are shown, with items presented in descending score order.

Among the suggestions to enhance care was the importance of early assessment, diagnosis, and treatment in enhancing CRPS care, alongside enabling earlier and easier access to psychological support for patients. However, effecting change for these factors was considered difficult; indeed, providing earlier and easier psychological support to patients was considered the least likely to be achievable of all the suggestions presented due to long waiting lists, lack of psychology services, and unclear referral pathways. Providing more education for therapists about CRPS and enabling them to know what to do when patients are not progressing was also reported as important. Furthermore, these actions were considered relatively easy to implement, alongside measures to ensure CRPS is explained to patients as early as possible and provide education to ‘other’ staff (including surgical staff) about CRPS.

When suggestions for a guidance document/resource pack were explored, case studies and troubleshooting examples were identified as most helpful. Psychological advice and relevant knowledge/education to share with patients was also felt appropriate for inclusion. Information about diagnosis, differential diagnoses, what to do when CRPS diagnostic criteria are not met, and an assessment checklist were suggested. There was a reported need for next steps advice, including local pathway options and available training courses, although subsequent discussion amongst stakeholder group attendees indicated that this level of information would be difficult to compile and challenging to keep up to date.

Additional ‘wish-list’ items generated by the findings in the qualitative interviews indicated a need to improve accessibility to patient information, with the provision of documents in a variety of formats and languages, as well as a need to have accurate patient-facing information returned as top results in internet searches. A desire for therapists to have access to advice from experts in the field was evident, and the potential highlighted for a CRPS forum/clinical support group/special interest group to be provided, aimed at those working outside specialist CRPS-practice services. A request for education for therapists, including experiential learning, was also expressed, as was a proposal that patient education should include ‘real-life’ experiences to which patients could relate.

## Discussion and conclusions

This work highlights the challenges of providing care for people with CRPS across existing pathways in England. By exploring the expressed needs and experiences of therapists working within and outside of specialist CRPS-practice services and capturing patient perspectives, opportunities and priorities to improve care were elicited.

Published guidelines from the Royal College of Physicians promote best practice in CRPS diagnosis, treatment and management in the UK and include recommendations for treatment approaches.^2^ It is notable that despite the existence of these comprehensive guidelines our findings highlighted a reported need for better CRPS management and for the provision of CRPS education and training. Furthermore, less than a third of respondents to our survey reported a defined management pathway for CRPS. The authors therefore suggest that there may be considerable value in future efforts to understand how to optimise the adoption and application of CRPS guidelines alongside other wider efforts to provide accessible training and education on the therapy techniques they recommend, in particular those techniques which are not part of usual therapy practice, for example, strategies to correct body perception disturbance.

Alongside medical management, care for CRPS, provided by either Physiotherapists or Occupational Therapists, has been found to improve physical function^19^ and to have a positive effect on pain reduction and symptom relief.^20,21^ Identifying and addressing the needs of therapists was therefore considered fundamental to improvements in future practice.

Whilst our data evidenced consensus about the importance of early diagnosis, assessment and treatment of CRPS, it also illustrated frustration that systemic challenges within services based outside of specialist CRPS-practice services (such as lack of available appointments, low staffing, time pressures, lack of access to psychological support, and complex referral pathways) prevented the achievement of these aims. Whilst similar findings have been reported internationally,^11^ stakeholders in the current study perceived that implementing change in these areas was difficult to achieve. Nevertheless, it is hoped that initiatives to improve musculoskeletal service provision, such as the publication, in 2024, of the Getting It Right First Time (GIRFT) Further Faster Community MSK services Handbook,^22^ will lead to improvements in access to appropriate and timely treatments within the NHS for people with CRPS.

What was considered to be impactful, and more feasible to achieve, would be to provide additional education to therapists and other clinical staff, as well as to people with early CRPS. Early patient education is strongly advocated by current guidelines for the management of CRPS^2,4,23^ and findings from a recent study by Lewis et al.^24^ have similarly suggested that tailored treatments aimed at improving the knowledge of people with early CRPS about their condition, would be beneficial in enhancing their understanding and providing a platform for better self-management. Patient perspectives highlighting the CRPS-related educational needs of healthcare professionals have been reported by Johnston-Devin et al.,^25^ and usefully, findings from a 2019/2020 International Delphi study have proposed 48 essential components for what CRPS education should comprise.^26^ Furthermore, a web application for CRPS has been recently launched with the support and endorsement of the European Pain Federation (EFIC); available from https://crps.europeanpainfederation.eu/. This resource provides comprehensive advice and education for clinicians and is available in multiple languages.

Another key finding of the current study was the reported desire by therapists for better personal access to experts in the field of CRPS and to be able to learn from their case studies or reports. This was also reflected in patients’ reports of feeling reassured by the knowledge and confidence of therapists working in specialist centres. A timely opportunity therefore exists for further initiatives to ensure the provision of case-study education and the sharing of specialist expertise and knowledge. This could be facilitated via professional bodies, or through the potential development of responsive forums hosted by special interest networks.

In considering the feasibility of the many suggestions to improve care, our stakeholder groups indicated that having core outcome measures to assist decision making regarding onward referrals would be relatively straightforward to provide to therapists. In recent years there has been an international effort to define and implement a CRPS core data set for research, with rigorous testing subsequently confirming these data are practical and feasible to collect.^4,27,28^ Adoption of this core data set will facilitate the establishment of an international research registry and data bank but may also be of value to therapists in supporting their clinical practice.

Both our survey and qualitative findings suggested the range of therapy-led treatments used for CRPS were largely consistent across settings, albeit dependent on available resources. This study did not set out to explore the efficacy of approaches; indeed, this has been the focus of other research.^4,29–32^ Nevertheless, our data highlighted a desire for better access to psychology input, as this was reported as rarely available outside specialist CRPS-practice services. The significant psychological impact of CRPS has been acknowledged and reported in the literature^33–35^; despite the reported support needs we identified in this current study, further research on the efficacy of psychological interventions for CRPS may endorse efforts to make psychology services more widely available, encourage the upskilling of other professional groups in providing this care, or facilitate the provision of online psychological support. Evidence from chronic pain studies has previously suggested enhanced effects from combining physiotherapy and psychological approaches, in comparison with physiotherapy alone^36^; similar research in CRPS may therefore be of value.

Of note, results from the interviews with healthcare professionals and the stakeholder workshops reflected the widely accepted assertion that early treatment of CRPS is fundamental to its management.^1,2,37,38^ Indeed, the introduction of a patient information leaflet, care management guidelines, and staff education about CRPS within a fracture clinic, plaster room, and Emergency Department of a regional hospital has been shown to reduce the incidence of CRPS after distal radius fracture.^38^ However, a recent analysis of clinical patient-reported outcome measures from a specialist multidisciplinary rehabilitation programme has challenged this opinion, finding that people with persistent CRPS maintained the improvement they gained during rehabilitation, whereas those treated with early CRPS did not.^24^ This apparent contradiction raises a potentially important consideration regarding the definition of ‘early’ CRPS. In the Lewis et al. study,^24^ ‘early’ was <1 year symptom duration, with outcomes reported for people already seen by a specialist CRPS-practice service. Conversely, therapists working outside of specialist CRPS-practice services may conceptualise ‘early’ differently, as they commonly see and treat people with symptoms of CRPS within the very first weeks after onset, and often before they have an established CRPS diagnosis. Further research is therefore needed to understand differing interpretations of ‘early’ CRPS and to seek to identify the optimum window for treatment.

A key strength of the current research was the multi-faceted nature of the programme of work and the breadth of participants recruited. Robust research methods were applied throughout, including a survey and interviews with therapists working with people with CRPS in a range of specialist and non-specialist settings. Importance was also given to the patient voice – considered essential to the improvement of future health care and treatment. Integrating the survey and interview data and applying a nominal group technique to identify the priorities for attention, has provided a clear roadmap for action, driven from the perspectives of those who best understand the condition, either from providing therapeutic care or living with CRPS.

The authors acknowledge the relatively modest size of the e-survey sample in the current study. In order to encourage respondents with relevant experience, we advertised the survey as being appropriate to physiotherapists and occupational therapists who ‘work, or have worked, with people with CRPS’. It is plausible that this inhibited responses from those who had only very limited experience of working in this area and who therefore did not feel confident to engage. It also precluded in the inclusion of podiatrists who we are aware may also be involved in the management of people with CRPS. A future survey with a more overtly inclusive invitation may encourage a greater response and comprehensive therapy representation.

Findings from the current programme of research have highlighted several avenues to directly inform and influence healthcare practice. Priorities for intervention, and their feasibility for action have been identified. The concept of a resource pack evolved from the data and provided a helpful framework for capturing these proposals. Whilst systemic concerns around care pathways are undoubtedly challenging to address, pragmatic suggestions were received for interventions focussed on education, support, and advice for therapists, and for the provision of patient information. These offer not only significant, and importantly achievable, opportunities to influence practice in the shorter term, they also provide a direction for future avenues of research, including the optimum time frame in which to provide this support.

## Data Availability

The datasets generated are not expected to be made publicly available due to the qualitative nature of the study and the capacity of the study team to ensure the complete dataset is fully anonymised prior to sharing. The study team do not have ethical approval or participant consent to share the dataset, only for extracted data to be anonymised and published. Individual requests to the study team to access the data will be considered.

## Note

1. Participant comments are annotated where P = patient, SP = therapist working within a specialist CRPS-practice service, and T = therapist working outside a specialist CRPS-practice service.
